# Community boundary spanners as an addition to the health workforce to reach marginalised people: a scoping review of the literature

**DOI:** 10.1186/s12960-018-0310-z

**Published:** 2018-09-10

**Authors:** Carolyn Wallace, Jane Farmer, Anthony McCosker

**Affiliations:** Swinburne University of Technology, Hawthorn, Victoria Australia

**Keywords:** Boundary spanning, Health services/utilisation, Marginalised, Community health workers, Navigators, Public health, Developed countries

## Abstract

**Background:**

Health services in high-income countries increasingly recognise the challenge of effectively serving and engaging with marginalised people. Effective engagement with marginalised people is essential to reduce health disparities these populations face. One solution is by tapping into the phenomenon of boundary-spanning people in the community—those who facilitate the flow of ideas, information, activities and relationships across organisation and socio-cultural boundaries.

**Methods:**

A scoping review methodology was applied to peer-reviewed articles to answer the question: “How do health services identify, recruit and use boundary spanners and what are the outcomes?” The review was conducted in seven databases with search terms based on community-based boundary spanning, marginalised people and health services.

**Findings:**

We identified 422 articles with the screening process resulting in a final set of 30 articles. We identified five types of community-based boundary spanning: navigators, community health workers, lay workers, peer supporters and community entities. These range from strong alignment to the organisation through to those embedded in the community. We found success in four domains for the organisation, the boundary spanner, the marginalised individuals and the broader community. Quantifiable outcomes related to cost-savings, improved disease management and high levels of clinical care. Outcomes for marginalised individuals related to improved health knowledge and behaviours, improved health, social benefits, reduced barriers to accessing services and increased participation in services. We identified potential organisational barriers to using boundary spanners based on organisational culture and staff beliefs.

**Conclusions:**

Community boundary spanners are a valuable adjunct to the health workforce. They enable access to hard to reach populations with beneficial health outcomes.

Maintaining the balance of organisational and community alignment is key to ongoing success and diffusion of this approach.

## Background

The purpose of this scoping review of the literature is to consider the role that might be played, in health service delivery and health literacy development in high-income countries, by community-based lay persons. Such people would be adjuncts to, and work with, existing health personnel. They would have features making them distinctly valuable, including deep community knowledge, innate networking skills, a mission for social benefits and low cost, or no cost—if they are volunteers. In a policy environment encouraging community capacity-building as a response to tackle health and social inequity, harnessing community-based boundary-spanning people (as we term them and explain later) could be an idea that is perfect for our time and contexts.

This review was prompted by an approach to us from a rural Australian public health service which sought to tackle long-standing healthcare engagement gaps of marginalised community members. The service had previously initiated several participatory activities, including a community-led healthy eating project and a community garden, to engage diverse community members in the available services, but noted an equity gap remained between socio-economic groups and uptake of services. Simultaneously, service providers had observed that some community members appeared to “naturally” cross social, cultural and organisational boundaries to forge links between different social groupings in the community. Staff wanted to know the extent to which other health services were harnessing such “boundary-spanning” community members to help reach marginalised community members and engage them in services. We define boundary spanning as facilitating “transactions and the flow of information between people or groups hindered by some gap or barrier” [1] (p.158) and community-based boundary spanners as people located in the community being served and acting as a boundary spanner both within the community and between the community and one or more health organisations.

We define marginalised people as those who are “socially excluded and experience inequalities in the distribution of resources and power” [2] (p.195). There is considerable evidence that marginalised people have poorer health status and outcomes [3]. While health services personnel may struggle to engage marginalised people, there exist community members who have both capability in accessing and using health services *and* relationships or connections with marginalised people in their community. The concepts of socio-cultural boundaries and boundary spanning are a useful way to frame this phenomenon.

Boundaries separate one group or one organisation from another [4, 5]. Symbolic and social boundaries also exist between social groups at a societal and community level [6]. They can confine people to marginalised groups—sometimes over generations, impacting on participation in community life [7] and access to services [8].

Boundary spanning describes the way some people bridge these organisational, symbolic and social boundaries. Considering organisations, boundary spanning occurs for several purposes, depending on context, including for accessing information [9], innovation and knowledge transfer [10], collaboration [8, 11] and for improving business performance [12]. Considering communities, boundary spanning occurs often as a form of social leadership [13–15]. Boundary spanners bring groups and individuals together for community advancement [14] and can draw on organisations’ resources to support local priorities. Community boundary spanners can bridge between organisations and communities as they operate and have relationships in both milieux [14, 16, 17].

In health, the term boundary spanning is used in health management literature. Boundary spanning is discussed for (1) *improving management and teamwork*—personnel are studied to assess the impact of their boundary-spanning management style on teamwork and staff satisfaction [18–21], (2) *care coordination*—for team members in areas including cancer care [22] and mental health [23], (3) *knowledge development and innovation*—encouraging interdisciplinary and cross-sector research [24–26], (4) *collective action with other sectors*—to address disadvantage, unemployment and community safety [27–29] and (5) *embedding practice change*—introducing new models of care including value-based care and patient safety practices [30].

With recent increased policy emphasis in developed country contexts on health services working more closely with their communities for collective benefit, (including on, for example, service integration, primary healthcare and co-design), an emergent challenge is identifying effective and cost-effective ways for staff and diverse community members to cross boundaries between institutions and community, to work together. The institutionalised nature of health—with health professionals set up as technical experts [8, 31]—presents a barrier for marginalised citizens who could receive positive health benefits from accessing services. Efforts to enhance access need to account for the symbolic and social boundaries that marginalised citizens’ experience, in addition to more obvious physical and institutional boundaries to accessing health services.

To find out if and how, community-based boundary spanners could be a useful adjunct to health workforce for engagement of marginalised citizens, we conducted a scoping review of the literature. The review was designed to answer the research question: “How do health services identify, recruit and use boundary spanners and what are the outcomes?” We sought four domains of insights—with respect to boundary-spanning people: for what purposes are they deployed; what ways do services work with them; their characteristics (so they can be identified); and outcomes for marginalised people of deploying them.

## Method

We used the Arksey and O’Malley [32] scoping review methodology with Levac et al. [33] enhancements. The methodology has five steps: identifying the research question; identifying relevant studies; study selection; data charting; collating, summarising and reporting the results.

This article’s authors compiled an initial list of search terms based on the *concept* of community-based boundary spanning, *population* of marginalised people, and *context* of health services. Terms were trialled initially by searching with Scopus and EBSCOhost and the terms “peer” and “excluded” were removed due to the high number of irrelevant articles they returned—such as peer reviewed and exclusion being used in descriptions of research methodologies.

The list of databases was developed in consultation with a topic librarian and by considering recent scoping reviews on similar topics—community participation [34] and patient navigation [35]. Databases searched were CINAHL, Scopus, PubMed, Medline, Health Business Elite, Health Source Nursing Academic Edition and Academic Search Complete. Table 1 lists search terms and inclusion and exclusion criteria. We included reviews as they are a form of research based on the analysis and synthesis of studies and provide additional background based on previous knowledge while still keeping within our search parameter of articles from 2007 to 2017. We extracted sufficient information from the reviews in the data charting process as summarised in Table 2.

**Table 1:** Search terms and inclusion and exclusion criteria

Key word	Search terms
Boundary spanning (concept)	“boundary spann*” OR “boundary cross*” OR “community guide*” OR “community aide” OR “community organi?er*” OR intermediary OR broker OR bridge* OR connector OR “translation agent” OR networker OR promatora OR navigator*
Marginalised (population)	Marginali?ed. OR disadvantage* OR “hard to reach”
Health services (context)	Hospital* OR “primary care” OR “community health” OR “health organi?ation*” OR “health service*” OR healthcare
Inclusion criteria	Article type: research, discussion, review or scoping review Addressing all three conceptual areas: health; boundary spanning; marginalised population About boundary spanning between health setting and the community About use of boundary spanners from the community context not the institutional setting—i.e. from the community or with similar attributes to community being served Located in high-income countries (added as criteria at stage three)
Exclusion criteria	Not in English Not peer-reviewed Published before 2007 Outlining a study that had not yet commenced

**Table 2:** Summary of scoping review findings

First author and year	Health context	Population group	Boundary spanning type	Paid or unpaid	Purpose of boundary spanning	Activities	Outcome domains			
							Individual	Boundary spanner	Organisation	Community
Aoun (2013) [61]	Weight loss	Rural middle aged to older men	Peer	Unpaid	Change in health behaviours and outcomes	Workshop/information sessions; individual provision of health advice/coaching	✓	✓		✓
Bailey (2015) [53]	General healthy lifestyle interventions	Prisoners and people with mental illness	Lay	Paid	Change in health behaviours and outcomes	Workshop/information sessions; individual provision of health advice/coaching; referrals to other organisations	✓	✓		
Barlow (2011) [54]	Perinatal/maternal health	Socio-eco deprived families	Peer	Paid and unpaid	Addressing organisational trust problem	Individual provision of health advice/coaching; home visits/phone calls; emotional/practical support; referrals to other organisations	✓	✓	✓	
Bustillos (2015) [38]	Nutrition	Disadvantaged and hard to reach	CHW	Paid	Addressing organisational trust problem	Workshop/information sessions; individual provision of health advice/coaching	✓	✓		
Choi (2016) [39]	Cancer screening or care	Ethnic minority	CHW	Unstated	Increase participation rates in clinical services	Workshop/information sessions; home visits/phone calls; service navigation	✓	✓		
Doolan-Noble (2013) [62]	Chronic care	Rural with complex and chronic conditions	Navigator	Paid	Solution to workforce shortage	Home visits/phone calls; emotional and practical support; referrals to other organisations; patient follow up and documentation	✓		✓	
Felix (2011) [45]	Mild to moderate functional limitations	Long-term care needs: age and disability	CHW	Paid	Increase participation rates in clinical services	Home visits/phone calls; referrals to other organisations; service navigation	✓		✓	
Gampa (2017) [46]	Community health	Indigenous communities	CHW	Paid	Solution to workforce shortage	Individual provision of health advice/coaching; home visits/phone calls; emotional and practical support; referrals to other organisations; cultural brokering; case management; health service provision		✓		
Han (2007) [47]	Chronic care	Older migrants	CHW	Unpaid	Change in health behaviours and outcomes	Workshop/information sessions		✓	✓	
Hartman (2013) [60]	Exercise program	Ethnic minority mothers	Community entities	Paid	Change in health behaviours and outcomes	Workshop/information sessions; cultural brokering	✓		✓	
Hesselink (2011) [27]	Perinatal/maternal health	Ethnic minority women	CHW	Paid	Change in health behaviours and outcomes	Workshop/information sessions; individual provision of health advice/coaching; cultural brokering; health service provision	✓		✓	
Kennedy (2010) [55]	Nutrition	Less affluent neighbourhood	Lay	Paid and unpaid	Change in health behaviours and outcomes	Workshop/information sessions; individual provision of health advice/coaching; emotional and practical support	✓	✓	✓	✓
Levinson (2015) [48]	Smoking cessation	Socio-eco disadvantaged	Lay	Paid	Increase participation rates in clinical services	Individual provision of health advice/coaching; home visits and phone calls; referrals to other organisations; patient follow up and documentation			✓	
Margellos-Anast (2012) [49]	Chronic care	Poor inner-city communities	CHW	Paid	Increase participation rates in clinical services	Individual provision of health advice/coaching; referrals to other organisations; case management.	✓		✓	
May (2007) [65]	Community health	Immigrants	CHW	Paid and unpaid	Solution to workforce shortage	Workshop/information sessions; individual provision of health advice/coaching; home visits/phone calls; emotional and practical support; referrals to other organisations	✓	✓	✓	✓
McLeish (2015) [56]	Perinatal/maternal health	Vulnerable mothers	Peer	Unpaid	Addressing organisational trust problem	Workshop/information sessions; individual provision of health advice/coaching; emotional and practical support; service navigation	✓	✓		
Najafizada (2015) [63]	Community health	Marginalised people	CHW	Paid and unpaid	Increase participation rates in clinical services	Workshop/information sessions; individual provision of health advice/coaching; health service provision	✓		✓	✓
Otiniano (2012) [50]	Community health	Hard to reach communities	CHW	Stipend for the workshop	Addressing organisational trust problem	Workshop/information sessions; community-based research		✓		
Palmas (2014) [51]	Chronic care	Hispanic adults	CHW	Paid	Change in health behaviours and outcomes	Individual provision of health advice/coaching; home visits/ phone calls; emotional and practical support	✓			
Palomino (2017) [52]	Cancer screening or care	Ethnic minorities- rural	Navigator	Paid	Increase participation rates in clinical services	Service navigation; cultural brokering.	✓			
Rafie (2015) [40]	Cancer screening or care	African-American	Lay	Unpaid	Increase participation rates in clinical services	Workshop/information sessions	✓	✓		
Raj (2012) [41]	Cancer screening or care	Racially and ethnically diverse	Navigator	Paid	Increase participation rates in clinical services	Home visits/phone calls; service navigation; patient follow up and documentation	✓			
Rohan (2016) [42]	Cancer screening or care	Medically disadvantaged	Navigator	Paid	Increase participation rates in clinical services	Home visits/phone calls; emotional and practical support; service navigation; patient follow up and documentation	✓			
Shahidi (2015) [43]	Community health	Inner city neighbourhood “forgotten”	CHW	Paid	Addressing organisational trust problem	Workshop/information sessions; home visits/phone calls; emotional and practical support; service navigation; case management; health service provision				✓
Sokol (2016) [64]	General healthy lifestyle interventions	Hardly reached	Peer	Paid and unpaid	Addressing organisational trust problem	Home visits/phone calls; emotional and practical support; service navigation; referrals to other organisations	✓		✓	
Thomson (2012) [57]	Perinatal/maternal health	Mothers in a deprived area	Peer	Paid and unpaid	Addressing organisational trust problem	Workshop/information sessions; home visits/phone calls; emotional and practical support	✓	✓		
Torres (2014) [67]	Perinatal/maternal health	New immigrants and refugees	CHW	Paid	Solution to workforce shortage	Workshop/information sessions; individual provision of health advice/coaching; home visits/phone calls; emotional and practical support; referrals to other organisations; cultural brokering; case management	✓		✓	
Wagoner (2015) [44]	Sexual health	Ethnic minority men- Latino	Peer	Unpaid	Change in health behaviours and outcomes	Workshop/information sessions; individual provision of health advice/coaching; emotional and practical support; referrals to other organisations	✓	✓		✓
White (2015) [58]	Mental health	High deprivation areas	Lay	Paid	Change in health behaviours and outcomes	Individual provision of health advice/coaching	✓			
Woodall (2013) [59]	Community health	Disadvantaged communities	Lay	Unpaid	Change in health behaviours and outcomes	Workshop/information sessions; individual provision of health advice/coaching; emotional and practical support	✓	✓		✓

**Fig. 1:**
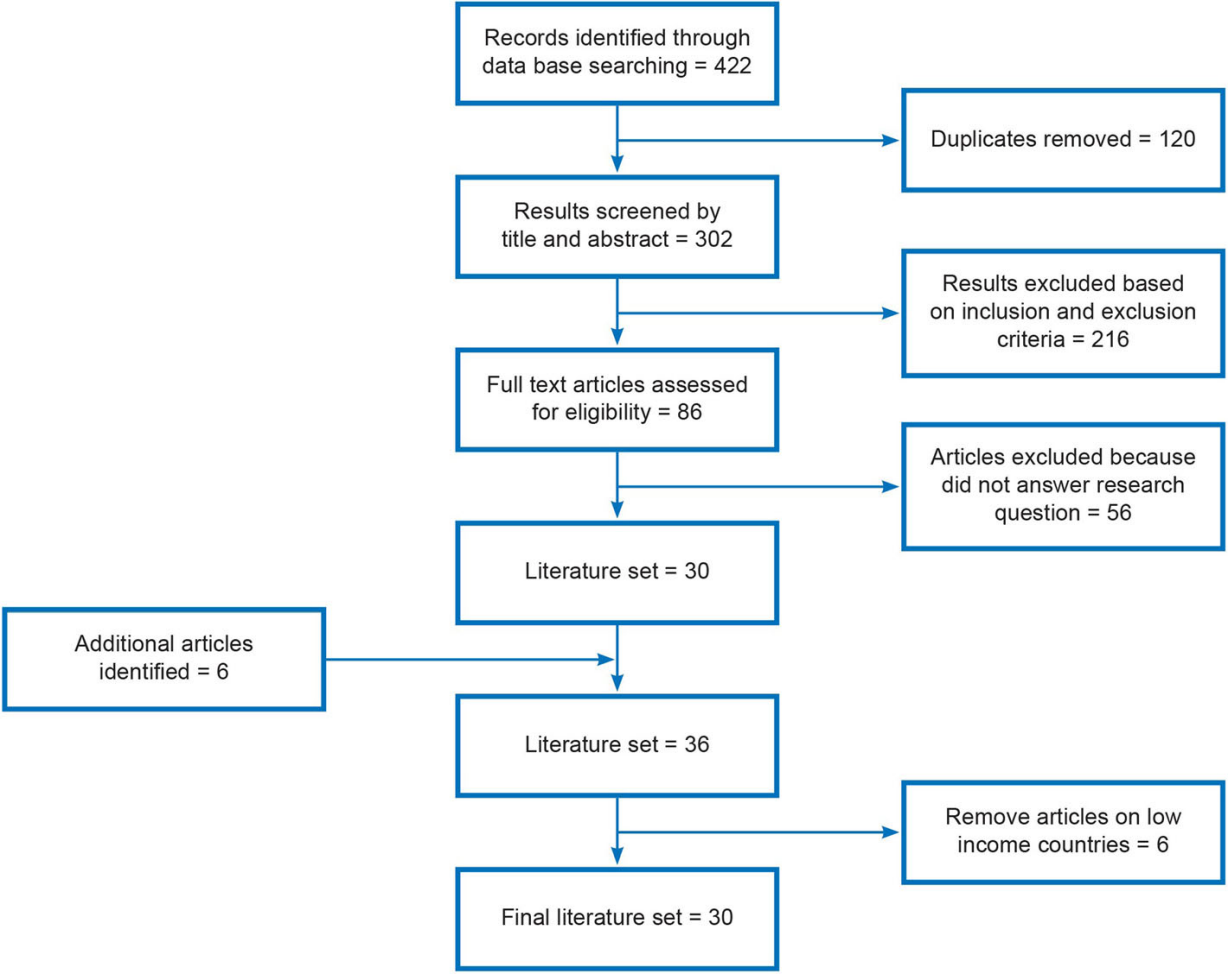
The search process

Figure 1 illustrates the search process. The initial 302 items were screened by one author (CW). CW read all full text articles and JF and AM each read half of this set. Where there was disagreement about eligibility, all three authors re-read the item and final inclusion was determined at a group meeting. Scoping reviews are an iterative methodology. Towards the end of the article selection process, the terms “champion”, “peer supporter” and “patient navigator” were added to the search due to their frequent use in the articles found. A final decision was made to refine the review to focus on high-income countries due to a distinction in the literature about use of community-based boundary spanners in lower- versus higher-income countries [36, 37].

Data charting was developed to enable description and analysis of the scoping review outputs. Initial charting was undertaken according to headings: study aim and design; health context and population; how boundary spanners were identified and recruited; training and support provided; qualifications, experiences and characteristics of boundary spanners; closeness to community; paid/unpaid tasks performed and outcomes. Secondary detailed charting focused on boundary spanners: title, paid/unpaid, prior health education or experience, live in the community, established networks, similarity to community being served, personal characteristics and where they interact with people. The data charting was undertaken by CW and verified with other authors.

## Findings

The findings commence with the health focus and countries where community boundary spanning is used and the descriptions and titles for boundary spanning. This is followed by factors relating to how and why health services use the boundary spanners, characteristics of the boundary spanners and outcomes from their activities. The section concludes with barriers to effective use of boundary spanners.

### Health focus and country

The health focus of boundary-spanning activities in the 30 articles was wide ranging (see Table 2). The majority were located in the USA (17) [38–52] or England (7) [53–59]. Two articles were from the Netherlands [27, 60], one Australia [61] and one New Zealand [62]. Two articles were reviews of high-income countries [63, 64].

### Descriptions, titles and types

The search did not produce any articles using the term boundary spanners for community-based people (in contrast with health management literature, as noted above). People fulfilling boundary-spanning roles in the community were described as “engaged insider” [43], “bridging role within the clinic” [54], “cultural bridge” [38, 41, 52, 59], “nexus” [46], “intermediary” [27], “connector” [48, 63, 65] “psychosocial bridge” [48] and “trusted liaison” [50].

We found the titles used to describe the boundary-spanning roles, such as “Patient Navigator”. “Champion” and “Health Coach” (Table 3) aligned with five fairly distinct *types* of boundary-spanning activity that emerged from the articles. These were navigators, community health workers, lay workers, peers and community-based entities. The navigator type emerged from a response to the complexity of cancer care with a focus on helping patients to overcome barriers to care [66]. Use of community health workers (CHW) originated in low-income countries to address shortages in local health workforce, using local people to provide basic healthcare services. Although community health workers are deployed in high-income countries, a recent review [63] notes that “there is no widely accepted definition of the concept for high-income countries” (p.e157). Lay workers are a non-clinical workforce [58] building on informal helping networks within a community [61]. In a similar vein, peer supporters act in a “non-professional capacity to offer support to others with whom they have some experience in common” [56] (p.258). Community entities refer to the community resources of the target group such as churches, community organisations, ethnic media, networks and events [60].

**Table 3:** Titles used for boundary spanners and the types they align with

Type of boundary spanner:	Navigators	Community health workers	Lay workers	Peers	Community entities
Boundary spanner titles	Patient navigator	Community health worker Promatora Community connector Community multicultural health broker Community health representative	Champion Lay food worker Community breast health advocate Health volunteer Natural helper Health trainer Smoking solution guide	Peer support Champion Health coach Lay health advisor Male lay health advisor Mentor Buddy Companion Community parent Community supporter	Ethnically specific channel
Number articles *n* = 30	4	13	6	6	1
Article reference	[41, 42, 52, 62]	[27, 38, 39, 43, 45–47, 49–51, 63, 65, 67]	[40, 48, 53, 55, 58, 59]	[44, 54, 56, 57, 61, 64]	[60]
	
	Organisational orientation • Technical • Service provision and access focus • Documented scope of practice • Alignment with mission and interests of health service			Community orientation • Not based in a health service • High degree flexibility • Relationship focus • Close knowledge of local community • Alignment with interests of the community	

These types have varying degrees of organisational through to community orientation. Organisational orientation is “the degree to which an individual’s behaviours are aligned with their own organization’s overarching mission, vision, and interests” (i.e. in this case, more oriented to the health institution). Community orientation is “the degree to which an individual is aligned with the interests of the community, a unified body of individuals with common interests, external to the [health] organization” [17](p.89) (more oriented to community). Thus the navigator type, recruited directly by the health organisation and in many cases a paid member of the organisation, has the highest organisational orientation and community entities, which are embedded in the communities they serve, have the highest level of community orientation.

The review revealed many articles discussing navigators and community health workers which were not included due to the person being an actual healthcare worker with no demonstrated membership or relational or geographical proximity to the community being served. These did not meet our definition of a community-based boundary spanner.

### Identification and recruitment

Twenty-one articles specified how boundary spanners were recruited. In eight, identification and recruitment were conducted along typical recruitment lines when the boundary spanner was expected to have a reasonably strong organisational orientation. There were 13 examples of identification and recruitment having a more community bottom-up, grass-roots approach. In these, boundary spanners were self-nominated, nominated by peers, or found through a community partner organisation.

### Purpose of boundary spanning

In all articles, the boundary-spanning roles were used to reach marginalised people with health benefits intended. The underlying rationale varied based on three issues—whether they were solving a workforce or organisational trust problem, focussed on improving the performance or uptake of health services, or focussed on engaging people in the community with each other and/or with health services (Table 2).

Where health services use community-based boundary spanners as a solution to a workforce shortage problem [46, 62, 65, 67], the boundary spanners are valued due to physical location in the marginalised community setting where it is difficult to recruit health workers. In the articles related to a connection or trust discrepancy between the health service and the community, their similarity to the community members being served is the reason for the health service using them [38, 43, 50, 54, 56, 57, 64].

Boundary spanning to improve performance and uptake of health services was used to increase participation rates in clinical services [39–42, 45, 48, 49, 52, 63] or to achieve a change in health behaviours and outcomes [27, 44, 47, 51, 53, 55, 58–61]. In the former, boundary spanners have a stronger organisational orientation, and in the latter, health services use boundary spanners because of their community orientation.

Eight articles showed boundary spanners used to connect marginalised people with health services and also to increase between-citizen connections within the community. These articles emphasised community cohesion and empowerment arising from boundary spanning, in addition to individual health benefits.

### Use of boundary spanners

The activities of boundary spanners (Table 2) reflect the purpose for which they are engaged. Where the main use is to extend the workforce, increase compliance with treatment/screening or improve the efficiency of the system, the tasks of boundary-spanning roles are practical and structured. When the health service is using the boundary spanners for community-based research, community strengthening or to provide support for behaviour change, boundary spanners have a broader range of tasks and functions that constitute community development, health promotion and advocacy [43, 55, 65].

### Training and supervision

Information about training and supervision was provided in 26 articles. Training intensity varied considerably, as did expectations of prior knowledge about health or the health system. Training was sometimes tailored as part of an intervention being trialled. Other programs had 1 day of training with refresher modules [61], some used a mix of face-to-face and on-line training [48]. Some used existing training programs such as the City and Guilds Health Trainer Qualification [53]. Other articles [38, 47, 50] discussed development and impact of training boundary spanners.

### Payment/non-payment

Whether boundary spanners were paid or not was significant to their deployment. The navigator model most favoured payment (i.e. in all four examples). The community health worker model mainly had payment for workers (nine out of 13 articles). Both navigator and community health worker models have a higher organisation orientation than other models, reflected in paid roles. Models that least used payment are the lay worker and the peer support models where the boundary spanner has a predominantly citizen support role and limited service delivery role.

### Characteristics

Proximity to the community being served is a defining characteristic of boundary spanners. All articles suggest use of boundary spanners because they are distinctive from other health workforce due to their proximity to the community or target group. This holds for boundary spanners that are paid and those volunteers. Other characteristics relate to personal traits, education and experience.

The characteristics of boundary spanners are often vaguely articulated. While navigators had some affinity for the community being served—either through local knowledge or as native language speakers, they had a strong organisational orientation and all roles described were located firmly within health service organisations, with varying community presence. Lived experience mattered in some cases. Two of the four navigator programs selected navigators with some prior health experience [42, 52], one deliberately recruited lay navigators [62] and it was not stated in the fourth example [41]. This demonstrates a varied application of a type that was initially designed for helping disadvantaged patients to use cancer screening and treatment.

Peer and lay worker roles show a high degree of closeness to community, with boundary spanners encouraged to engage their family and social networks for their health promotion activities or to extend their community-based networks through their role. The predominant characteristics of the peer and lay roles are trusted, supportive, empathetic and non-judgemental. As the titles suggest, the lay and peer boundary spanners were valued for their closeness to community and willingness to work with citizens. Only two of six lay examples noted lay workers had prior health knowledge or experience [40, 53] and none of the six peer examples mentioned prior health knowledge or experience as a requirement.

Community health workers were expected to live in the community in all but one instance [27]. Trust, respect and supportiveness were dominant character traits for the community health workers. There were two examples with a community led approach to determining the important characteristics for community health workers, by the community being served [43] or the cohort of community health workers [67]. The community health worker examples did not rely on prior health knowledge or experience, with the exception of one article where the worker was integrated into a health practitioner team [27].

When an organisation rather than an individual was approached for its boundary spanning, the organisation had spokespersons who were local and trusted leaders from the ethnic community with well-established networks and no particular health knowledge or expertise [60].

### Outcomes

The outcomes reported from health services using community-based boundary spanners were all positive, although the research designs were varied and often relatively weak. Some articles described pilot studies [48, 49], while others reviewed one or more existing programs to determine outcomes [47, 50, 55, 65]. The majority used qualitative methodology (16); ten had mixed methods and four used solely quantitative methods. Quantifiable outcomes were net cost savings to Medicaid spending and decreased use of nursing home services [45], improved knowledge and management of childhood asthma [49], a non-significant trend towards improvement in clinical markers of diabetes [51] and high levels on quality indicators in cancer care [41].

Outcomes were found in four domains, for the organisation or system, boundary spanner, individual community member (social, mental and physical), and community collectively. Only two articles considered outcomes in all four domains [55, 65], while five considered outcomes in three domains [44, 54, 59, 61, 63]. Some articles did not consider outcomes for individuals or the community but focussed on other outcomes; for example, impacts of training or organisational traits on boundary-spanning people [40, 47, 50, 65].

Outcomes for marginalised individuals were described in 26 articles. Outcomes were positive and related to health [38, 40, 44, 49, 51, 53, 55, 58, 59, 61, 63, 64], social benefits [42, 52–59, 65], reduced barriers to accessing services [52, 62, 67] and increased participation [27, 39, 41, 45, 48, 60]. The majority of health outcomes were not clinical but related to health knowledge and behaviours.

An outcome of using lay people as boundary spanners in health settings is the potential health benefits for the boundary spanners themselves. Fifteen articles noted outcomes for the boundary spanners. Most common was increased confidence and knowledge which sometimes led to the boundary spanners expanding their role to include advocacy [38, 44, 47] or progressing to further education or employment [59]. Boundary spanners found that acting as a role model prompted improvements in their physical and mental health [47, 53, 59, 61]. Two articles [56, 65] report negative impacts for boundary spanners caused by tension between competing expectations of the host health organisation and the community they belonged to.

There were examples of boundary spanners having a wider impact on health services and systems through issue identification and advocacy [54, 65, 67]. Twelve articles report specific outcomes for organisations or the wider health system. These were cost savings [45, 49, 63], assisting staff with workload and a positive work environment [27, 47, 54, 62], practice change [65, 67] and enabling contact with hard-to-reach clients [55, 60, 64, 65].

Only seven articles described outcomes for the wider community where the boundary-spanning activity was occurring. These were more tentative and were predominately concerned with improved health knowledge and behaviour [55, 59, 61, 63, 65], social benefits of increased community competency [61, 65], reduced social isolation [65]; identification of community needs [43, 44, 65] and improved social cohesiveness [59].

### Barriers

A small number of articles noted potential barriers to the success of deploying boundary spanners. In a USA setting, Felix et al. [45] found concerns about the potential “woodwork effect” and its increased costs to Medicaid from community connectors encouraging citizens to “come out of the woodwork” and take up services to which they were entitled. One of the three sites in a case study of Turkish community health workers in the Netherlands was not as effective as others because midwives were reluctant to support special culturally tailored programmes [27]. In another case, the staff of a children’s centre were initially concerned with privacy issues of community members accessing service data when a peer support service was introduced [54]. Doolan-Noble et al. [62] describe “patch protecting” behaviour of health professionals who felt threatened by new patient navigators encroaching on their professional scope of practice.

## Discussion

The literature shows that a range of boundary-spanning roles have been trialled in high-income countries with the intention of engaging marginalised community members in health services and that there is some evidence of good outcomes. These roles are thus successfully acting to extend health workforce teams in spaces that are problematical for established institutionalised health system models. The essential feature underpinning success of community-based boundary spanning is deploying those with genuine closeness to the community being served. This requires identifying and recruiting boundary spanners for their location, shared experience and compatible demographic characteristics. Health knowledge is not essential and in most cases not expected. Training, supervision and ongoing support appear to be features of successful deployment. Organisational investment in boundary-spanning roles varies, linked to the extent of health expertise desired and integration in health practitioner teams.

The evidence we found suggests that community-based boundary spanning, as a strategy to engage marginalised people, could be further exploited in high-income countries’ health systems. The majority of examples we found show boundary spanners used instrumentally with tasks focusing on: providing support (practical and emotional), education and information, service navigation and service referral. Many of the boundary-spanning roles valued by service providers are those offering cultural interpretation or bridging mechanisms for provision of services as they currently exist; however, there are few examples of genuine support for boundary spanners as agents of community empowerment and activation or as system challengers.

Despite the evidence we uncovered that using community-based boundary spanners has potential for engaging marginalised people, it appears there can be reluctance from staff to accommodate these roles into health teams. This leads us to suggest that a significant barrier to greater implementation of boundary-spanning roles in health relates to organisational and workforce culture. Using community-based boundary spanners requires health service personnel to cross boundaries in their own thinking and attitudes about professional and lay workers (or unpaid volunteers), working in teams—for, and with, communities.

The findings from this review indicate that, if considering boundary-spanning roles, health services need to decide where these will operate on the spectrum from an organisational to community orientation. An organisational orientation requires supervision, training and a degree of integration with teams, systems and working practices. Community orientation is the crux of this model’s success, so community orientation must remain central. If health services are prepared to move beyond a merely instrumental use of community-based boundary spanners, evidence suggests this model has potential for enabling structural changes and increasing community health literacy, service access and wider collective capacity benefits.

One notable surprise for us was the lack of appearance, in the literature, of use of digital technologies as part of community-based boundary-spanning activities. Despite digital technologies being a commonplace tool for contemporary communication [68], only one article [40] included an example of a boundary spanner using social media to reach marginalised people. This might be particularly useful to reach dispersed communities in rural areas.

Based on our review, we suggest that future research might focus on systematically measuring the outcomes of different boundary-spanning models, testing opportunities to use digital technologies in boundary spanning and strategies for more systematic deployment and diffusion of this community-boundary-spanning phenomenon.

## Conclusion

In health management literature, boundary spanning is an established term and proven strategy for improving the way health organisations function and collaborate with partner organisations to improve systems and health outcomes. In literature of health service provision and public health, we found that, although health services use several types of community boundary-spanning roles to improve the health of marginalised people in the community, the term boundary spanning is not used to describe the same fundamental phenomenon. There is a significant literature on community-based boundary-spanning roles, but they have a range of names, including navigators, community health workers, lay workers and peer supporters. Some boundary-spanning work is conducted through community organisations.

We conclude there are opportunities to understand and socialise how boundary-spanning works by discussion in health teams and to further develop boundary-spanning roles to realise opportunities for engagement between health services and communities. Both “sides” would benefit from this as a policy direction for health systems to address health inequalities and increase community participation. To embed the adoption of community boundary spanners into delivery of health services, such a policy framework should ensure (a) allocation of health personnel time to recruit and work with boundary spanners, (b) inclusion of boundary-spanning roles in health service planning and provision and (c) resources to train and support community boundary spanners. Further deploying boundary-spanning roles will depend on careful management of the tensions placed on boundary spanners who need to maintain their community identity while also working with the health service and for health staff who may fear erosion of their professional roles and expertise by lay workers.
